# Young-onset dementia: scoping review of key pointers to diagnostic accuracy

**DOI:** 10.1192/bjo.2019.36

**Published:** 2019-06-04

**Authors:** Mary O'Malley, Jacqueline Parkes, Vasileios Stamou, Jenny LaFontaine, Jan Oyebode, Janet Carter

**Affiliations:** Research Assistant, Faculty of Health and Society, University of Northampton, UK; Professor, Faculty of Health and Society, University of Northampton, UK; Research Assistant, Centre for Applied Dementia Studies, University of Bradford, UK; Research Fellow, Centre for Applied Dementia Studies, University of Bradford, UK; Centre for Applied Dementia Studies, University of Bradford, UK; Assistant Professor, Division of Psychiatry, University College London, UK

**Keywords:** Young onset dementia, diagnosis, assessment, review, accuracy

## Abstract

**Background:**

Routine psychiatric assessments tailored to older patients are often insufficient to identify the complexity of presentation in younger patients with dementia. Significant overlap between psychiatric disorders and neurodegenerative disease means that high rates of prior incorrect psychiatric diagnosis are common. Long delays to diagnosis, misdiagnosis and lack of knowledge from professionals are key concerns. No specific practice guidelines exist for diagnosis of young-onset dementia (YOD).

**Aims:**

The review evaluates the current evidence about best practice in diagnosis to guide thorough assessment of the complex presentations of YOD with a view to upskilling professionals in the field.

**Method:**

A comprehensive search of the literature adopting a scoping review methodology was conducted regarding essential elements of diagnosis in YOD, over and above those in current diagnostic criteria for disease subtypes. This methodology was chosen because research in this area is sparse and not amenable to a traditional systematic review.

**Results:**

The quality of evidence identified is variable with the majority provided from expert opinion and evidence is lacking on some topics. Evidence appears weighted towards diagnosis in frontotemporal dementia and its subtypes and young-onset Alzheimer's disease.

**Conclusions:**

The literature demonstrates that a clinically rigorous and systematic approach is necessary in order to avoid mis- or underdiagnosis for younger people. The advent of new disease-modifying treatments necessitates clinicians in the field to improve knowledge of new imaging techniques and genetics, with the goal of improving training and practice, and highlights the need for quality indicators and alignment of diagnostic procedures across clinical settings.

**Declaration of interest:**

None.

Young-onset dementia (YOD), arbitrarily described as dementia diagnosed under 65 years, is poorly recognised and often misdiagnosed.^1,2^ This clinical review evaluates the current evidence about best practice in diagnosis to guide thorough assessment of the complex presentations of YOD.

Many clinical practice guidelines on the diagnosis and management of dementia exist, but vary in the grading systems used to assess the quality of evidence and the strength of recommendations.^3^ There are currently no specific practice guidelines about diagnosis in YOD although excellent practice pointers specific to key areas are available.^4–6^ The differential diagnosis of YOD encompasses complex presentations of the common primary neurodegenerative diseases as well as autoimmune, inflammatory, late-onset metabolic and hereditary/familial causes.^5^ Although Alzheimer's disease makes up the majority of cases, it represents a significantly smaller percentage than in late-onset Alzheimer's disease (LOAD) and presentations are frequently non-amnestic in nature.^7^

## Heterogeneity of presentation in YOD

Dementia diagnosis is dominated by the traditional view, derived from the numerical dominance of LOAD, that all dementia is associated with episodic memory loss and functional decline. For some clinicians in general practice, adult psychiatry and older adult memory services, atypical presentations of the common dementias in younger people are rarely encountered and this can result in delay to referral, clinical underinvestigation, misdiagnosis and delays in obtaining a definitive diagnosis.^8,9^

For example, one in three people with young-onset Alzheimer's disease present with problems associated with posterior cortical atrophy (PCA). Rather than reporting memory problems, that are more typical of LOAD,^10^ those with PCA experience problems with object recognition and other visual changes. Autosomal dominant forms of frontotemporal dementia (FTD) and Alzheimer's disease are also common and may present with neurological symptoms.^11^ People with behavioural variant FTD (bvFTD), may start to show lack of empathy or concern for others, and social disinhibition, such as being overfamiliar with strangers or acting on aggressive or appetitive urges, by swearing or overeating sweet foods.^12^ Changes in managing complex tasks may be identified by individuals becoming apathetic, perseverative or failing to plan ahead. Similarly, those with primary progressive aphasias^13^ are likely to experience various problems with language, for example retaining meaning of words, producing or finding words. None of these difficulties are well-captured through most of the common cognitive screening tests for dementia, as these are focused predominantly on testing orientation and memory.

## Biological differences in YOD

In addition to issues of heterogeneity, emerging evidence confirms distinct biological differences between LOAD and young-onset Alzheimer's disease, with the latter having greater neocortical pathology, particularly in the parietal cortex, greater tau compared with amyloid burden, and less hippocampal disease.^11^ Recent analysis suggests that an age cut-off of 70 provides better differentiation on neuropsychology testing between early- and late-onset Alzheimer's disease than the standard cut-off of 65 years old.^14^ There is also emerging evidence of younger people with bvFTD having significantly higher rates of disinhibition, more loss of sympathy/empathy and more perseverative, compulsive behaviours compared with those with late onset.^15^ Thus, the evidence suggests that effective diagnosis of YOD must be guided by differences that distinguish it from late-onset disease, and also encompass the inherent heterogeneity within a younger population.^16^

## Delays to diagnosis and misdiagnosis

Evidence regarding delays to diagnosis has identified that although 60% of young-onset patients sought help within 12 months of symptom onset,^17^ it took an average of 3.3 years in a young-onset Alzheimer's disease group and 4.9 years in a young-onset FTD group to receive a formal diagnosis.^18^ More recent studies, indicate that the average time to diagnosis was 4.4 years in younger people for all-cause dementia compared with 2.2 years for late-onset disease of comparable severity.^19^ Increased time to diagnosis for younger people, is more likely when the younger person receives a diagnosis of FTD, rather than other dementia types.^20^ Given the significance of changes in empathy and disinhibition often associated with FTD, delay in diagnosis can mean that close relationships break down prior to diagnosis or that people take considerable risks. Additionally, the time to dementia diagnosis is significantly longer when the dementia is other than Alzheimer's disease or FTD.^21^ The INSPIRED (Improving Services for Younger Onset Memory and Related Disorders) study from Australia recently reported time to final diagnosis of the type of dementia from first presentation as 4.7 years.^20^ Participants with younger onset experienced significantly longer time to first consultation and to family awareness of the dementia diagnosis.^22^

## Psychiatric presentation

Atypical presentations of YOD frequently overlap with psychiatric conditions resulting in misdiagnosis as a psychiatric illness preceding final diagnosis of YOD, accounting for 28% of individuals presenting to a specialist clinic in a retrospective masked chart review study.^23^ The consequences of misdiagnosis include delay to diagnosis, use of ineffective and potentially harmful treatments, delays to getting appropriate support and increased family stress.^24^ Furthermore, several studies^1,2,25^ have evaluated the quality of the diagnostic work-up in patients aged 65 years and younger using evidence-based guidelines for the diagnostic evaluation of dementia as reference standards including as a minimum: history of cognitive symptoms, cognitive testing, psychiatric evaluation, physical examination including neurological examination, assessment of activities of daily living, a battery of blood tests, electrocardiogram and computed tomography or magnetic resonance imaging (MRI) scan of the brain. An acceptable diagnostic work-up including all items of recommended basic diagnostic evaluation was performed in only 24%.^1^

The aim in this review is to inform the debate regarding the essential elements of the diagnostic process in YOD, over and above the use of current diagnostic criteria for disease subtypes and to identify current advances and research findings in preparation for an international Delphi consensus on diagnosis of YOD funded by the Alzheimer's Society. It is not intended to provide an overview of key features and assessment of the main neurodegenerative conditions that are covered comprehensively elsewhere.^6,26–29^

## Method

A comprehensive search of the literature, adopting a scoping review methodology^30^ was conducted, and updated in September 2018. Two electronic search engines were used: PubMed and Web of Science. We focused only on research articles that had been published in peer-reviewed journals to ensure the evidence base and methodology used was rigorous. The databases were selected as they particularly cover life science and biomedical fields of research and allowed us to focus on whether our key search terms were found in the highlighted papers' abstracts.

### Search strategy and selection criteria

Systematic and concise terms were used to search for relevant papers (see Appendix 1 for details of the terminology). The terms groups were combined using Boolean operators, using the AND function in the following manner: 1 AND 2 AND 3.

#### Additional inclusions

The initial search was conducted on 16 June 2017, to include papers published between the years 2012 and 2017. On 12 September 2018 the search was re-run and updated using the original search terms to establish if any additional papers had been published; therefore, the search included papers published between 16 June 2012 and 12 September 2018. Studies conducted internationally (i.e. outside of the UK) were included, although only if the articles were written in English. Database search strategies were supplemented with snowballing methods,^31^ including reference list and citation searches, author searches and hand searching of key journals.

#### Exclusion criteria

The exclusion criteria were as follows.

- Studies focused on Korsakoff/alcohol-induced dementia or dementia caused by HIV/AIDS, as these conditions traditionally have different pathways to diagnosis and care.
- Dementia research solely focused on late-onset dementia, as these would not reflect YOD.
- Articles concerning late-onset dementia.
- Papers related to psychosocial approaches and basic neuroscience.
- Articles focused solely on qualitative reports about the experience of assessment and diagnosis from people living with YOD.

Two reviewers (J.C. and M.O.M.) independently screened titles and abstracts identified by the search and applied the selection criteria to potentially relevant papers. The full texts that were appropriate and included in the review following initial abstract screening were then read in full by the same two authors.

### Data extraction

**Fig. 1:**
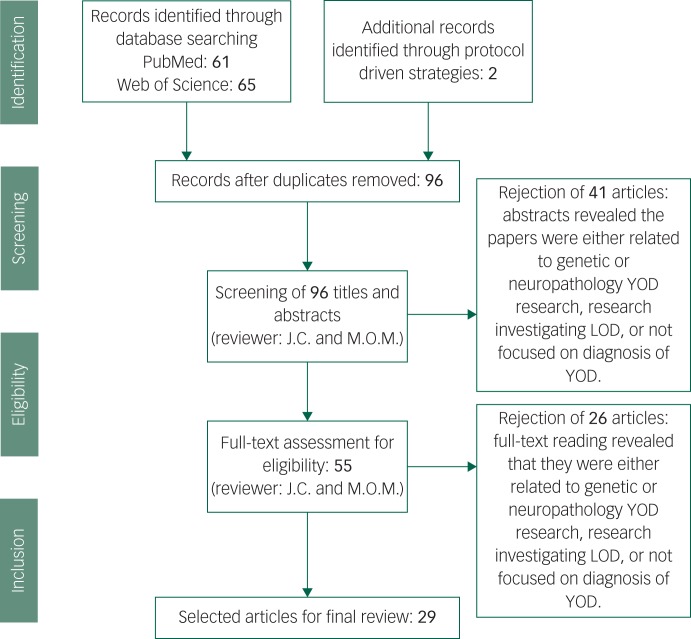
Screening and literature selection procedure.

YOD, young-onset dementia; LOD, late-onset dementia.

Our data extraction process involved removing duplicated papers from the two separate databases, including any additional papers found through protocol-driven strategies. All abstracts were reviewed for whether they met criteria, and full texts were read for those remaining papers. After reading the 55 full-texts, 23 articles were rejected as it was discovered upon reading that they did not meet our exclusion criteria. In total 29 papers met criteria and were included (see Fig. 1).^1,2,11,14,20,23,25–29,33–35,42,46,47,51,52,54,58,60,62,64–68,70^

## Results

The 29 papers were grouped into themes related to the standard clinical approach to diagnostic assessment, i.e. history taking, physical examination, investigation and diagnosis. A summary of key pointers for the clinical assessment is outlined in Appendix 2.

### History taking

The majority of papers related to traditional methods of clinical history taking (see Appendix 2). However, Snowden *et al*, identified that a guided, more structured approach to diagnosis in identifying Alzheimer's disease and FTDs in younger people leads to higher correlation with post-mortem findings.^33^ In this approach, consideration of time course of the illness, pattern of physical, behavioural and cognitive symptoms, comparison of anterior versus posterior cognitive deficits and specificity of cognitive deficits, for example non-focal or focal, yielded high specificity and sensitivity (FTD 97% and 100%, respectively; Alzheimer's disease 100% and 97%, respectively) where supplementary neuroimaging had been unhelpful. Within this broad framework, the importance of taking a collateral history from an informant/family member was generally emphasised^27,28,32,33^ but felt to be particularly salient in those presenting with non-cognitive behavioural problems or personality change that might indicate a diagnosis of FTD.

When eliciting key symptoms, most research has focused primarily upon discrimination of FTD from Alzheimer's disease. Key areas, namely, stereotypic or ritualistic behaviour (such as clockwatching, stereotyped use of catch-phrases, preoccupation with counting and numbers), appetite increase and change in preference for sweet food, disinhibition, and features of poor social awareness have been shown to reliably separate groups with FTD from those with Alzheimer's disease on factor analysis of a carer-rated scale of neuropsychiatric symptoms for assessment of patients where conventional cognitive tests are unlikely to be discriminatory.^54^ The suggested use of open-ended questions during history taking such as ‘has the patient said or done anything embarrassing?’, ‘does he or she seem indifferent/obsessive or less affectionate?’ may help elucidate these key symptoms.^26,27^ For Alzheimer's disease, detailed understanding of key features may help. For example, in the absence of a reliable family history, a retrospective cohort study of familial and non-familial early-onset Alzheimer's disease with the commonest *PSEN1* mutation identified the following clinical characteristics as key features that may aid discrimination; an early and progressive age at onset, history of headaches, myoclonus, gait abnormalities and pseudobulbar affect.^34^

### Family history

A history of familial disease is particularly relevant in YOD as familial forms of neurodegeneration are more frequent in this age group. Taking a clear family history of dementia or other neurological diseases is advocated, in particular the use of a detailed family history of at least three generations.^26^ High risk, indicating the need for genetic testing, may be indicated by a history of one affected relative in the case of FTD^35^ and two first-degree relatives with early-onset Alzheimer's disease in the Alzheimer's disease.^53^ One should note that this information may be insufficient for those who lack a known or reliable family history and be masked in those with small families and premature death because of other causes. A comprehensive overview of approaches to genetic testing and counselling in YOD is beyond the scope of this review and is available elsewhere.^35^

### Psychiatric history

Psychiatric evaluation can identify behavioural and psychological symptoms that are common and contribute to patient distress and care burden.^1^ However, very limited attention has been given to the importance of psychiatric assessment and development or use of appropriate tools despite clear evidence of frequent presentation of younger people living with dementia (YPD) to psychiatric services. High rates of psychiatric misdiagnosis are driven by the significant overlap in symptoms of neurodegenerative disease especially bvFTD and psychiatric disorders.^55^ In one study, 28% of individuals with a neurodegenerative disease had a prior incorrect psychiatric diagnosis.^23^ Across groups, depressive disorders and bipolar affective disorder were the most common misdiagnoses although a diagnosis of schizophrenia was not uncommon. Rates ranged from <12% in those with atypical presentations such as prominent language, speech or movement disorders and up to 52% in those with bvFTD.^23^ Over 50% of patients waited up to 3 years before the diagnosis was revised. Although onset in 30 s and 40 s may overlap with age at onset for a psychotic disorder, lack of first-rank symptoms and a clear family history, often autosomal dominant in nature, of a neurodegenerative condition should raise suspicion of a neurodegenerative condition.

Younger age at symptom onset, limited education and a strong family history of psychiatric illness are significantly associated with prior psychiatric misdiagnosis in bvFTD. The key features at presentation identified by clinicians as suggesting a functional rather than organic aetiology were emotional symptoms and eating symptoms often leading to misdiagnosis of those with FTD as having major depressive disorder.^23^ Equally, apathy, social withdrawal and lack of initiation may be mis-identified as depression.^56^ As aids to discrimination, the ritualistic and impulsive behaviours of bvFTD usually lack the anxiety of obsessive–compulsive disorders, and dietary changes such as overeating with dramatic weight gain are uncharacteristic of depressive disorders. Similarly, history of longstanding delusions, command auditory as opposed to visual hallucinations, intermittent history of anxiety and depression, hypomanic episodes and distressing compulsions impacting on day-to-day life are more indicative of primary psychiatric illness.

The use of a structured carer-based questionnaire such as the Neuropsychiatric Inventory can be helpful to capture changes in behaviour and to assist diagnostic accuracy.^39^ Neuropsychiatric symptoms have a significant impact on patients and supporters and represent the main predictor of a move to institutional care.^57^

Recently, discovery of the *C9orf72* mutation associated with bvFTD has focused upon psychosis as a presenting psychiatric symptom in FTD, which may occur years before dementia onset.^53^ Psychotic symptoms are not included in current diagnostic criteria for FTD. A recent review, based on 122 publications, concluded that the approximate prevalence of psychotic symptoms in FTD is 10%.^55^ Among those with a known genetic background, psychotic symptoms have been found to be especially common in both progranulin and *C9orf72* carriers with reported prevalence of 25% of progranulin mutation carriers^58^ and around 50% or more in *C9orf72*.^59^ Misdiagnosis of patients carrying the *C9orf72* mutation with a psychiatric diagnosis (e.g. schizophrenia) is reported and this may be particularly problematic for those patients with no neurological signs to orientate diagnosis.

Overall, the evidence suggests that a high index of suspicion is necessary for younger and mid-life patients who present with new-onset depressive, behavioural, psychotic or cognitive symptoms.^23^ Additional assessment pointers to avoiding misdiagnosis as psychiatric illness include re-evaluation of previously diagnosed treatment-resistant depression that may be a proxy for comorbid dementia.^20^

### Physical and neurological examination

No specific guide to neurological examination in YOD is available in the literature but good clinical practice would suggest that thorough neurological examination accompanied by physical examination is a vital part of the approach to accurate diagnosis. Although, typically unremarkable, early identifying clues to possible diagnoses may include apraxia, parkinsonism, upper and lower motor neuron symptoms, eye signs, cerebellar signs, extrapyramidal signs and frontal release signs.^5,6,32^

### Investigations

#### Neuropsychology and cognitive assessment

Neuropsychology testing acts as a gatekeeper to more extensive investigations and provides objective evidence of cognitive deficit. Generally, more subtle approaches to cognitive assessment are required in YOD and measures such as the Montreal Cognitive Assessment^43^ and Addenbrookes Cognitive Examination^44^ may be unhelpful. More accurate assessment may be enhanced by the use of appropriate assessment tools. For example, there is increasing recognition that bvFTD may have an amnestic component, making it more difficult to distinguish from Alzheimer's disease, despite diagnostic guidelines. Several studies indicate that in the absence of cerebrospinal fluid (CSF) biomarkers and amyloid positron emission tomography (PET) imaging, social cognition tools (such as, the (Mini) Social Cognition and Emotional Assessment Tool) may be more useful in distinguishing bvFTD from Alzheimer's disease.^41^ In addition, the Frontal Assessment Battery^45^ that tests for a number of cognitive changes associated with FTD (conceptualisation, mental flexibility, motor programming, sensitivity to interference, inhibitory control and environmental autonomy) may be useful for eliciting clinical symptoms.

Recent studies support a differentiated pattern of neuropsychological impairment in some dementia subtypes according to age. For example, Palasi *et al* showed that patients with young-onset Alzheimer's disease performed worse than those with LOAD in attentional, imitation praxis and verbal learning tests, and that an age cut-off of 70 differentiated between early- and late-onset groups better than the standard cut-off of 65 years old.^14^ These findings emphasis that scoring thresholds may need to be adapted on routine neuropsychological test batteries when administered to younger adults.

Furthermore, despite current diagnostic criteria for FTD, at least in the early stages, the traditional view of episodic memory impairment in young-onset Alzheimer's disease and language and executive dysfunction in FTD may not be a useful discriminatory factor and a more comprehensive assessment is necessary.^46,47^ In a large comparison of neuropsychological data in pathologically confirmed cases of Alzheimer's disease and FTD, the neuropsychological test battery of the Uniform Data Set,^60^ which is essentially a standard test battery, with the exception of memory, did not separate cognitive performance across the two groups. This raises the issue of whether in the absence of specific biomarkers, standard neuropsychological test batteries, used frequently in memory clinics, contribute to accurate diagnosis and indicate that more tailored approaches are essential. Many tests of cognitive impairment are used in memory clinics without having been validated in populations under 65.^61^

#### Biomarkers

##### Neuroimaging

Neuroimaging remains a crucial first-line investigation in diagnosis of all types of dementia. MRI is considered the preferred modality to aid differential diagnosis or exclude diagnoses often by aiding assessment of patterns of atrophy.^29,62^ Advances in techniques and image analysis increasingly support the inclusion of more specialised imaging protocols as a key biomarker in diagnosis of YOD although translation into use in everyday clinical practice is lacking. Medial temporal lobe atrophy is recognised as a supportive biomarker for the diagnosis of Alzheimer's disease. The medial temporal lobe atrophy (MTA) scale is the commonest visual rating scale of hippocampal atrophy in Alzheimer's disease and its use extends to clinical practice in some settings.^63^ The cut-off used for the scale is critical to discriminate disease from ‘non-cases’. However, for Alzheimer's disease in younger populations, where presentations with PCA are more common, the Posterior Atrophy scale in combination with the MTA scale may aid discrimination.^64^ It may also have a role to discriminate Alzheimer's disease from FTD and enhance diagnosis of focal Alzheimer's disease presentations in a younger group where MTA may be absent.^7^

An analysis of three different visual rating scales looking at sensitivity and specificity of MTA cut-off scores suggested that adjustment of cut-offs according to age would help further with diagnostic accuracy in younger populations.^63,64^ However, more clinically realistic, as such sophisticated techniques are generally only available in major centres, visual inspection by an experienced neuroradiologist has equally been demonstrated to be highly correlated with pathologically confirmed diagnosis.^48^ Similarly, normal MRI in cases of clinically severe dementia should prompt reconsideration of diagnosis.

##### Clinical use of molecular imaging

The growing evidence base for molecular imaging has led to the introduction of clinical guidelines^65^ recommending the use of fluorodeoxyglucose (FDG)-PET or single-photon emission computer tomography (SPECT), principally, for all individuals with early-onset cognitive decline and for prominent non-amnestic presentations involving language, visuospatial, behavioural executive and/or non-cognitive symptoms in Alzheimer's disease or prominent amnestic presentation in non-Alzheimer's disease dementias. Although only a few studies are available with evidence about specificity and sensitivity of FDG-PET and SPECT in atypical Alzheimer's disease, the European federation of the Neurological sciences recommend their use when diagnosis is in doubt after structural imaging and clinical work-up.^4^ Similarly the use of amyloid PET imaging is advocated in young-onset Alzheimer's disease presenting with non-amnestic symptoms, in patients presenting with clinically atypical presentations, and in differentiation of Alzheimer's disease from FTD as the latter is not associated with amyloid deposition in younger patients.^4,66,67^ Additionally, the guidelines suggest that there is value in functional imaging in those with severe cognitive impairment in psychiatric disease or where cognitive testing is not possible.

##### CSF biomarkers

Three types of biological marker are found in CSF and are currently in use: Aβ1–42 amyloid protein, total tau (T-tau) and phosphorylated tau (P-tau), tau proteins. Although many published studies suffer from a lack of masking and pathological confirmation of diagnosis, the lower frequency of mixed pathology in younger people and the reduced likelihood of pathological change in CSF compared with older patients has led to the suggestion that positive Alzheimer's disease CSF biomarkers may be most useful in diagnosis of YOD.^51^ The National Institute of Ageing–Alzheimer's Association criteria for Alzheimer's disease dementia recognises the importance of positive Alzheimer's disease CSF biomarkers in research diagnostic criteria guidelines but suggests that challenges in achieving required sensitivity and specificity, and centre-to-centre variability, precludes their use in clinical practice. Conversely, the Alzheimer's biomarkers standardisation initiative^52^ reached consensus that Alzheimer's disease CSF biomarker analysis be considered as a routine clinical test in all early-onset dementia, atypical or complex presentations. Given the variation in attitudes and use of CSF biomarkers, it has been suggested that developing consensus guidelines on CSF-related methodologies and how they are applied clinically would be beneficial.^68^ The new National Institute for Health and Care Excellence guidelines in 2018 outline the value of FDG-PET or perfusion SPECT and/or CSF biomarkers in a systematic approach to identifying specific dementia subtypes.^69^

There is currently no conclusive head-to-head comparison study of amyloid PET imaging in early versus late-onset disease,^70^ and no firm evidence regarding a hierarchy of implementation with regard to CSF markers versus amyloid PET^71^ to guide clinical practice. ‘Appropriate use’ criteria for amyloid PET imaging in patients with progressive dementia and atypically young age^49,50^ support its value.

## Discussion

Prevalence figures for the numbers of people with YOD in most countries (including the UK) are lacking, presenting a significant hurdle to adequate provision of specialist services. Patient groups continue to express concerns about long delays to diagnosis, lack of knowledge from key professionals, lack of continuity of care and limited information at the time of diagnosis.^72^ Equally, because the majority of dementia services remain primarily focused upon the needs of older people, gaining access to age-appropriate post-diagnosis interventions remains challenging for younger people.^73^

YOD presents a significant diagnostic challenge and atypical presentations and overlap with psychiatric syndromes are common. This complexity often results to delays in diagnosis and additional stress, frustration and burden for families. The evidence confirms that YPD see on average a minimum of two and some up to five different specialists before receiving a final diagnosis and pathways into care in the UK are chaotic.^73^ Typically, many YPD have lost their jobs before the opportunity for reasonable adjustments and vocational rehabilitation in the workplace and there are attendant economic costs of unnecessary appointments, potentially ineffective treatments and loss from the work force of family members.

Providing an accurate diagnosis is the first stage in allowing families access to treatment and support and help reduce uncertainty about the future. The majority of younger people in the UK continue to be assessed and diagnosed in mental health-led memory clinics where limited access to other disciplines is well documented.^73^ Furthermore, clear advice on a best practice approach to ascertaining diagnosis in younger patients is lacking. This raises concern as many dementia/memory clinics continue to employ routine procedures, screening measures and cognitive tests tailored to older patients that are often insufficient to identify the complexity of presentation in YPD and result in underinvestigation with limited use of crucial supplementary investigations. Indeed, evidence suggests that underinvestigation is particularly common in non-specialist settings. For example, a large study comparing over 5000 patients with LOAD and YOD demonstrated that extended investigations including extensive cognitive evaluation by a neuropsychologist, language assessment by a speech therapist, structural brain imaging with MRI, lumbar puncture with analysis of dementia biomarkers in CSF, electroencephalography or functional brain imaging with SPECT/ PET were generally required to reach diagnosis in the young-onset group.^25^

With this in mind, improving recognition and knowledge of YOD for primary care physicians and non-specialists where such facilities may be scarce must be an important goal. A new decision-making tool developed by the Young Dementia Network UK has been specifically designed to guide diagnosis and raise awareness of key red flags to diagnosis.^74^ Equally, ensuring access to training and demonstration of key competencies should be a key consideration for such services. In regional, rural, and remote areas, initiatives to expand consultation services using videoconferencing and telementoring has been demonstrated to be a valuable tool and could be envisaged for complex case discussion and imaging reviews.^75^

The evidence presented here supports the view that a multidisciplinary/multispecialist assessment within a specialist YPD service or centre is necessary for establishing YOD diagnosis and integration between specialists and partnership with a broad range of services (including third sector) is vital to help connect patients and their families with support at home and in their community. This approach is emphasised in the view from professional bodies.^76^ Facilitating consultation with experts more widely to those in rural areas who have limited access to key diagnostic tools is a vital part of this outreach in order to ensure alignment of process across services. Identifying a minimum standard for accuracy in diagnosis is likely to be helpful in this regard.

Furthermore, given the substantial burden of assessment outlined, consulting YPD about how best to undertake assessment and diagnosis would further guide good practice, and user and family organisations should be supported to participate in policy making and service planning. This approach overlaps with the best practice model of care developed by the Young Dementia Network UK which is supported by key stakeholders^77^ and forms the basis of current research to improve diagnosis for YPD (http://www.ucl.ac.uk/psychiatry/the-angela-project).

### Strengths and limitations

A scoping literature search identified the latest advances regarding diagnosis of dementia in younger people. Evidence was sparse and mostly involved expert opinion pieces and key practice pointers in the field. The limitations of the current evidence base include the potential bias of information from experts depending on speciality and the lack of controlled clinicopathological studies employing trials of clinical guidelines already in use for dementia in younger populations. Limited information is available about standard approaches to assessment of YOD in mental health services where understanding key elements of best practice would be valuable.

## Implications

The quality of evidence identified in this review is variable with the majority provided from expert opinion and evidence is lacking on some topics. Evidence appears weighted towards diagnosis in FTD and its subtypes and young-onset Alzheimer's disease. Accurate diagnosis is crucial in allowing individuals to understand and manage life with dementia at any age. For YPD, the diagnosis is ‘out of time’ and associated with specific and unique needs. This review highlights the importance of undertaking a comprehensive and patient-specific approach to diagnosis of YOD and is intended to assimilate emerging information from new fields for clinicians. The literature demonstrates that a clinically rigorous and systematic approach is necessary in order to avoid mis- or underdiagnosis for younger people. The review is not intended to be a comprehensive systematic review, but to provide a guide to psychiatrists and others in the field about current thinking. With the advent of new disease-modifying treatments there is an obligation to upskill clinicians in new imaging techniques and genetics, with the goal of improving training and practice, and highlights the need for quality indicators and alignment of diagnostic procedures across clinical settings.

## Appendix 1

**Table:** A breakdown of the three criteria (defining terms for young-onset, individual dementia diagnoses and terms related to the diagnostic process) and terms used when searching the search engines for appropriate articles

Group	Terms (in title or abstract)
1 Defining terms for young onset	TS: ((‘young onset’ OR ‘younger onset’ OR ‘early onset’ OR ‘presenile’ OR ‘working age’ OR ‘YOD’ OR ‘under 65’) NOT (‘elderly’ OR ‘older’ OR ‘late’))
2 Defining terms for dementia, and individual diagnoses	TS: (‘dementia’ AND ‘Alzheimer's’ OR ‘vascular dementia’ OR ‘frontotemporal dementia’ OR ‘Semantic dementia’ OR ‘Huntington's disease’ OR ‘acquired brain injury’ OR ‘Parkinson's disease’ OR ‘Creutzfeldt-Jakob’ OR ‘CJD’ OR ‘Lewy bodies’ OR ‘Picks disease’ OR ‘cognitive impairment’ OR ‘neurocognitive disorder’ OR ‘Posterior Cortical Atrophy’)
3 Defining terms for the diagnostic process	TS: (‘diagnosis’ AND ‘assessment’ OR ‘diagnostic’ OR ‘GP’ OR ‘misdiagnosis’ OR ‘misdirection’ OR ‘referral’ OR ‘clinicians’ OR ‘biomarkers’ OR ‘neuropsych’ OR ‘neuroimage’)

TS, Searching the Topic field.

## Appendix 2

**Table:** Key points: red flags for diagnosis

Clinical assessment	Consider potential red flags in	Description and references
History taking	Presenting complaints	Detailed history taking from a family member/informant is essential^27,28,32,33^ Ask about key emotional, cognitive and behavioural symptoms^33^ Identify anterior and posterior deficits^33^ Key features that may aid discrimination in familial disease; an early and progressive age at onset, history of headaches, myoclonus, gait abnormalities and pseudobulbar affect^34^
	Family history	Ask about first-degree relatives who have a diagnosis of a neurological condition or dementia^35,36^ Take a detailed family history of three generations^26^
	Psychiatric history	Consider use of a rating scale to assess mood and behaviour for example, Geriatric Depression Scale,^37^ Hamilton Rating Scale for Anxiety and/or Hamilton Rating Scale for Depression^38,39^ or the Neuropsychiatric Inventory (NPI)^40^ Caution when ascribing emotional and eating symptoms to mood disorders
	Behavioural symptoms	Consider: (a) use of social cognition questionnaire^41^ and (b) use of NPI^40^ Beware late-onset psychosis and treatment-resistant depression as a proxy for YOD^20^
Physical examination	Neurological examination	Look for: apraxia, parkinsonism, upper and lower motor neuron symptoms, eye signs, cerebellar signs, extrapyramidal signs, frontal release signs^5,6,32^
Mental state examination	Psychiatric assessment	Consider carer-based questionnaires including NPI Consider red flags to discriminate functional from organic cause^12,23,26,42^ Ask about: mood; appetite change; sleep; compulsive behaviour/rituals; abnormal beliefs/perceptions; personality change
Investigations	Neuropsychology Cognitive tests	Consider tests of decision-making, emotion processing and social cognition test in cognitive assessment in FTD Addenbrookes Cognitive Examination-III, Montreal Cognitive Assessment, Frontal Assessment Battery^43–45^ Consider formal neuropsychology testing in all individuals with suspected YOD in those under 70^46,47^ Use validated tools
	Neuroimaging magnetic resonance imaging	Visual inspection by an experienced neuroradiologist of medial temporal atrophy^48^
	Amyloid positron emission tomography	Consider for all early-onset dementia, and atypical or complex presentations Consider ‘appropriate use’ criteria^49,50^
	Cerebrospinal fluid analysis (CSF)	Consider CSF lumbar puncture for all early-onset dementia, and atypical or complex presentations^51,52^ Consider ‘appropriate use’ criteria^49,50,69^
	Genetic testing access to symptomatic and predictive testing	In FTD and Alzheimer's disease Consider testing in symptomatic individual with: (a) early-onset Alzheimer's disease/FTD in the setting of a family history of dementia; (b) autosomal dominant family history of dementia with one or more individuals with young-onset Alzheimer's disease or FTD; (c) a relative with known mutation^35,53^

YOD, young-onset dementia; FTD, frontotemporal dementia.
